# Evaluation of the adeno-associated virus mediated long-term expression of channelrhodopsin-2 in the mouse retina

**Published:** 2009-08-21

**Authors:** Elena Ivanova, Zhuo-Hua Pan

**Affiliations:** Department of Anatomy and Cell Biology, Wayne State University School of Medicine, Detroit, MI

## Abstract

**Purpose:**

The conversion of inner retinal neurons to photosensitive cells via viral mediated expression of channelrhodopsin-2 (ChR2) offers a new potential approach for the restoration of vision after photoreceptor degeneration. This study was conducted to evaluate the recombinant adeno-associated virus serotype 2 (rAAV2)-mediated long-term expression and safety of ChR2 in the mouse retina.

**Methods:**

rAAV2 vectors carrying a fusion construct of channelopsin-2 (Chop2) and green fluorescent protein (GFP; Chop2-GFP) under the control of a hybrid cytomegalovirus early enhancer and chicken β-actin (CAG) promoter were injected at different concentrations into the eyes of wild-type adult mice. The retinas were harvested up to 18 months after virus injection for immunostaining and electrophysiological studies. Injected mice were kept either under normal light conditions, or exposed to a strong blue light. The expression of GFP and the density of the cells in the ganglion cell layer (GCL) were examined.

**Results:**

The expression of Chop2-GFP was stable for up to 18 months. Chop-GFP was observed predominantly in retinal ganglion as well as amacrine cells. At the highest virus concentration (6×10^12^ GC/ml), up to 20% of the cells in the GCL were infected by the virus. At the lowest virus concentration (1×10^10^ GC/ml), the expression was targeted to AII amacrine cells. The concentration of the virus, the light conditions, and the percentage of Chop2-GFP-positive cells had no effect on the density and, thus, on the survival of the cells in the GCL. Sufficient number of functional ChR2 channels were maintained in ganglion cells to drive robust membrane depolarization and spike firing in response to light.

**Conclusions:**

Expression of Chop2-GFP could be achieved in retinal neurons in vivo for the duration of the lifespan of mice. The expression of Chop2-GFP did not cause any detectable toxicity and cell death to neurons of the ganglion cell layer.

## Introduction

The expression of microbial type rhodopsins, such as algal channelopsin-2 (Chop2), offers a potential strategy for the restoration of vision after photoreceptors have been lost in retinal degenerative diseases [1,2]. Channelrhodopsin-2 (ChR2), which refers to Chop2 with an attached chromophore, are directly light-gated channels that are permeable to physiological cations [3]. These channels have been expressed in various mammalian tissues in vitro and in vivo without toxicity to the cells (in the retina [1,2,4,5]; in the hippocampus [6,7]; in the primary somatosensory cortex [8]; in a transgenic mouse line [9]). However, most of the studies of ChR2 expression rely on cultured cells, short-term expression, or light conditions in which the ChR2 were not activated for long.

Viral-based gene transfer is a promising tool for the delivery of transgenes to non-dividing mammalian neurons. Previous studies have reported the ability of ChR2 expression in inner retinal neurons in vivo through the use of recombinant adeno-associated virus (rAAV) vectors [1,4,5]. To evaluate its potential clinical applications, we investigated the effects of virus concentration, light condition, and duration of expression on the long-term stability of ChR2 in mouse retinal neurons.

## Methods

### AAV vector injection

All of the animal experiments and procedures were approved by the Institutional Animal Care and Use Committee at Wayne State University and were in accordance with the NIH Guide for the Care and Use of Laboratory Animals. The injection of the rAAV, serotype 2 (rAAV2) that carried a fusion construct of Chop2 and green fluorescent protein (GFP; Chop2-GFP) under the control of a hybrid cytomegalovirus early enhancer and chicken β-actin (CAG) promoter has been previously described [1]. Briefly, C57BL/6 mice (purchased from the Jackson Laboratory, Bar Harbor, ME), age 1–2 months, were anesthetized by intraperitoneal injection of a mixture of 120 mg/kg ketamine and 15 mg/kg xylazine. Under a dissecting microscope, a small perforation was made in the temporal sclera region with a needle. A total of 1 μl viral vector suspension in saline was injected into the intravitreal space through the hole with a Hamilton syringe. Viral vectors were packaged and affinity purified at the Penn Vector Core in the School of Medicine Gene Therapy Program at the University of Pennsylvania (Philadelphia, PA).

After injection, animals were kept in the animal facility under normal housing conditions with food and water ad libitum and the room light intensity of 6x10^14^ photons/cm^−2^s^−1^, measured at a wavelength of 500 nm. One month after the viral injection, one group (n=4) of the animals continued to stay under the normal light condition. A second group (n=8) was exposed to a blue light (1.2×10^16^ photons cm^−2^s^−1^) for either 12 h (n=4), 1 week (n=2), or 2 weeks (n=2). Both light conditions were on a 12 h:12 h light-dark cycle. The blue light was generated by high power LEDs with a peak wavelength of 455 nm. The retinas of the light-exposed animals were examined two weeks after the blue light exposure.

### Immunocytochemical staining

The expression of GFP in the retina was examined in retinal whole-mounts and vertical sections. Mice were deeply anesthetized with CO_2_ and decapitated. The retinas were fixed in the eyecups with 4% paraformaldehyde in 0.1 M phosphate buffer (PB) for 20 min. The retinas were cryoprotected in a sucrose gradient (10%, 20%, and 30% w/v in PB, respectively). Cryostat sections were cut at 20 μm. For the whole-mounts, the retina was dissected free in PB solution, flat mounted on slides, coverslipped, and viewed under a Zeiss Apotome microscope (Zeiss, Oberkochen, Germany).

In most cases, GFP fluorescence was not enhanced with antibodies. When GFP fluorescence was enhanced with antibodies, it will be referred to as immunofluorescence. For immunostaining, retinal sections or whole-mounts were blocked for 1 h in a PB solution that contained 5% Chemiblocker (Chemicon, Temecula, CA), which is a membrane-blocking agent, 0.5% Triton X-100, and 0.05% sodium azide. The primary antibodies were diluted in the same solution and applied overnight followed by secondary antibodies for two hours. All steps were performed at room temperature. The primary antibodies were rabbit anti-GFP antibody conjugated to Alexa488 (1:2,000; Molecular Probes, Eugene, OR) and rabbit anti-Disabled-1 antibodies (1:8,000; Dab1; Chemicon, Temecula, CA). The secondary antibodies for Dab1 were donkey anti-rabbit Alexa594 (1:600; Molecular Probes).

**Figure 1:**
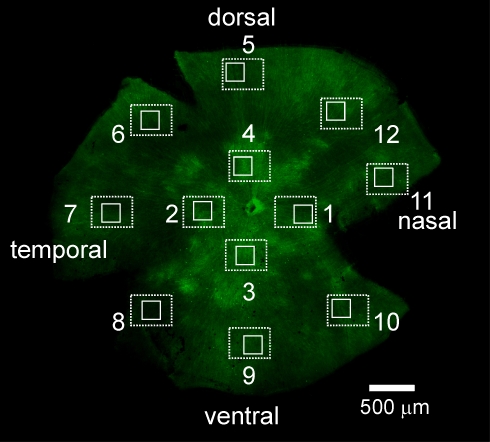
Representative fluorescent image shows Chop2-GFP expression in retinal whole-mount preparation. Retinas were photographed in 12 standard areas of 450×335 μm^2^ (dashed rectangles). The number of cells in the GCL was counted in characteristic smaller areas of 0.04 mm^2^ shown by solid rectangles (200×200 μm^2^).

The density of the cells in the ganglion cell layer (GCL) was estimated based on 4',6-diamidino-2-phenylindole (DAPI) staining [10,11]. Retinal whole-mounts were incubated for 20 min in the same solution used for immunostaining plus 5 μM DAPI. The tissue was rinsed in PB, flat-mounted, and viewed under a microscope. To estimate the density of the cells in the GCL, we photographed DAPI-stained retinas in 12 standard areas of 450×335 μm^2^ (Figure 1, dashed rectangles), and counted the cell numbers in characteristic smaller areas of 200×200 μm^2^ (0.04 mm^2^; Figure 1, solid rectangles). The numbers of the cells were averaged from several eyes (n) and shown as mean±standard deviation. Like other investigators, we did not count endothelial cells, which could be clearly identified by their elongated shape and close association with blood vessels, and astrocytes. The astrocytes have very small cell bodies with dense nuclei and are slightly displaced toward the optic fiber layer [12]. Thus, our numbers represent a mixture of ganglion and displaced amacrine cells.

### Patch-clamp recordings

Retinal slices were prepared as previously described [13]. Briefly, retinas were dissected free in oxygenized extracellular solution and mounted on a filter paper (Schleicher and Schuell, Dassel, Germany) with photoreceptor side up. Vertical slices of 150 μm thickness were cut together with the filter with a blade and maintained at room temperature. Photoreceptor cell-mediated light responses were bleached under our experimental conditions due to the use of the bright light during the slice preparation and the finding of GFP-positive cells for recordings. The recording chamber was superfused with oxygenized extracellular solution, which contained the following ingredients: 136.8 mM NaCl, 5.4 mM KCl, 0.4 mM KH_2_PO_4_, 0.3 mM NaH_2_PO_4_, 1.3 mM CaCl_2_, 0.5 mM MgCl_2_, 0.5 mM MgSO_4_, 5.0 mM HEPES, 22.0 mM D-Glucose, and 0.03 mM phenol red, pH 7.2. The intracellular solution contained 140.0 mM K-gluconate, 7 mM KCl, 0.1 mM EGTA, 4.0 mM MgCl_2_, 10 mM HEPES, 2.0 mM NaATP, 0.5 mM NaGTP, and 0.1 mM Alexa568 hydrozide sodium salt, pH 7.4 (Molecular Probes). Liquid junction potential (approximately 10 mV) was corrected offline. The resistance of the electrode was 7–9 MΩ. The series resistance, which was usually less than 40 MΩ, was left uncompensated. Pipette and cell capacitances were canceled. Data were analyzed offline using the Origin (Microcal Software, Northampton, MA) program.

### Multi-electrode array recordings

The multi-electrode array recordings were previously described [1]. Briefly, the retina was dissected and placed photoreceptor side down on a piece of nitrocellulose filter paper (Millipore Bioscience Research Reagents, Temecula, CA). The mounted retina was placed in the MEA-60 multi-electrode array recording chamber of 30 μm diameter electrodes spaced 200 μm apart (Multi Channel System GmbH, Reutlingen, Germany), with the ganglion cell layer facing the recording electrodes. The retina was perfused at 34 °C in oxygenated extracellular solution, which contained the following: 124 mM NaCl, 2.5 mM KCl, 2 mM CaCl_2_, 2 mM MgCl_2_, 1.25 mM NaH_2_PO_4_, 26 mM NaHCO_3_, and 22 mM glucose, (pH 7.35 with 95% O_2_ and 5% CO_2_). D(−)-2-Amino-5-phosphonopentanoic acid (D-AP5), L-(+)-2-Amino-4-phosphonobutyric acid (L-APB), and 6-Cyano-7 nitroquinoxaline-2,3-dione (CNQX) were purchased from Sigma-Aldrich (St. Louis, MO).

The duration of the light stimulation was 1 s and the interval between successive light stimuli was 60 s. The signals were filtered between 200 Hz (low cutoff) and 20 kHz (high cutoff). The responses from individual neurons were analyzed by using Offline Sorter software (Plexon, Inc., Dallas, TX).

### Light stimulation

In whole-cell patch-clamp recordings, light stimulation was generated by a 175 W xenon-based lamp (Lambda LS, Sutter Instrument, Novato, CA) and coupled to the microscope through an optical fiber. A band-pass filter of 420–490 nm was used to optimize the light spectrum (Nicon B-3A filter). For the multi-electrode array recordings, light stimuli were generated by a scanning monochromator with a 10 nm bandwidth. The light intensity was attenuated by neutral density filters.

## Results

### Long-term stability of Chop2-GFP expression in retinal neurons

**Figure 2:**
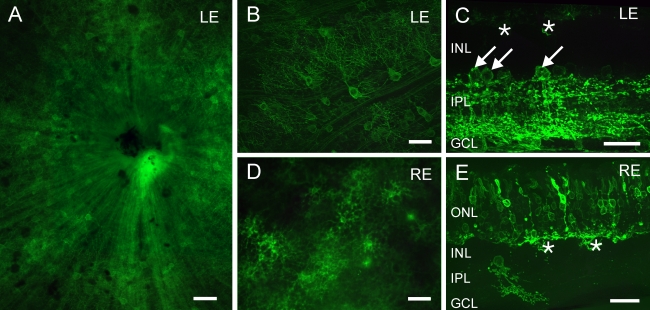
Long-term expression of Chop2-GFP in different retinal cell types. The images were taken from the retina of a 20-month-old mouse that was injected with the virus at the age of 3 months. In the left eye (**A**, **B**), numerous ganglion cells can be seen in the whole-mount. In addition, AII amacrine cells (arrows) and horizontal cells (asterisks) were visible in a vertical section of the same retina (**C**). In the right eye (**D**, **E**), many horizontal cells that express the Chop2-GFP construct were found in the whole-mount (**D**). In the vertical section, photoreceptors and horizontal cells (asterisks) showed GFP fluorescence (**E**). In **C** and **E**, GFP fluorescence was enhanced with antibodies against GFP. Abbreviations outer nuclear layer (ONL); inner nuclear layer (INL); inner plexiform layer (IPL); ganglion cell layer (GCL). Scale bars equal 50 μm in **A**, **B**, and **D**, and 25 μm in **C** and **E**.

To assess the long-term stability of Chop2-GFP in retinal neurons, we examined the expression of GFP 18 months after virus infection at a concentration of 1x10^12^ genome copies (GC)/ml. Similar to the results described with shorter durations after the injection [1], Chop2-GFP expression was usually confluent throughout the retina. The expression was predominantly observed in the GCL. The axons of the individual GFP-positive ganglion cells were clearly seen at optical nerve head in all retinas (Figure 2A). In a higher magnification (Figure 2B), the morphology of the cell bodies, dendritic trees, and axons were evident. In vertical sections (Figure 2C), numerous GFP-immunofluorescent cells in the GCL projected their processes to the entire inner plexiform layer (IPL). In addition, amacrine cells were observed in the inner nuclear layer (INL; arrows). Most of these amacrine cells in the INL were found to be AII amacrine cells (see next section). However, GFP-positive bipolar cells were rarely observed and GFP-positive astrocytes or Muller cells were not encountered. The morphological properties of all the GFP-positive cells appeared normal. We did not detect any abnormal swellings of the GFP-positive cells.

In a few cases, the Chop2-GFP was found to be predominantly expressed in distal neurons. In such cases, numerous GFP-fluorescent horizontal cells were observed in retinal whole-mounts (Figure 2D). In vertical sections, many photoreceptors exhibited GFP immunofluorescence in the inner segment, soma, axon, and axon terminal but not in the outer segment (Figure 2E). The morphology of the GFP-immunofluorescent photoreceptors also appeared normal. When Chop2-GFP was present in photoreceptors, it was detected only in a few inner retinal neurons (Figure 2E). The expression of Chop2-GFP in photoreceptors was likely caused by an unintended injection into the subretinal space. Infection of photoreceptors after subretinal injection with AAV2 in the mouse retina has been reported previously [14-16].

Together, these results indicate that long-term expression of Chop2-GFP can be achieved in inner retinal neurons as well as in photoreceptors for up to 18 months.

### Expression of Chop2-GFP does not cause neurotoxicity in retinal neurons

**Figure 3:**
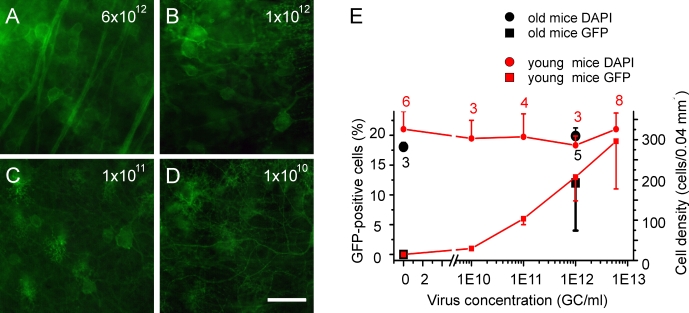
Dependence of the infection rate on the virus concentration. **A**-**D**: Representative images of 200×200 μm^2^ squares show the retinas that were infected with four different virus concentrations. **E**: The averaged percentages of GFP-positive and DAPI stained cells were plotted against the virus concentrations for the young adult mice (GFP, red squares; DAPI, red circles) and for old mice (GFP, black squares; DAPI, black circles). The numbers of investigated retinas are shown in red digits for young mice and in black digits for the old mice. Scale bar equals 50 μm in **A**-**D**.

We next examined whether the expression of Chop2-GFP could cause any neurotoxicity to retinal neurons. We chose to assess the neurotoxicity of the neurons in the GCL, which contains both ganglion and amacrine cells. We first examined the effect of virus concentration on the infection rate of the neurons in the GCL. Most of the mice in this set of the experiments were killed 1–2 months after the viral injection and they will be referred to as young adult mice. Some of the mice were kept for up to 18 months after the viral injection and the latter will be referred to as old mice. Figure 3 shows representative images of retinas from the young adult mice that were infected with four different virus concentrations. At the highest virus concentration used in this study, 6×10^12^ GC/ml, GFP fluorescence was observed in 20.5%±6% of the cells in the GCL. The percentage of GFP-positive cells decreased with the virus concentration. At the lowest concentration (1×10^10^ GC/ml), only a few GFP-positive cells were detected in the GCL (Figure 3D). The relationship between the virus concentration and the percentage of GFP-positive cells in the GCL is shown in Figure 3E (red squares). For the old mice that were injected with 1×10^12^ GC/ml of virus, the percentage of GFP-positive cells was similar to that of the young adult mice that were injected with the same virus concentration (Figure 3E, black squares).

The cell density of DAPI-stained cells (Figure 3E, red and black circles) was not affected by the viral infection and the expression of Chop2-GFP in both old (*t*-test, p=0.08) and young (one-way ANOVA, p=0.3) adult mice. These results indicate that the expression of Chop2-GFP does not cause cell death under normal light conditions.

**Figure 4:**
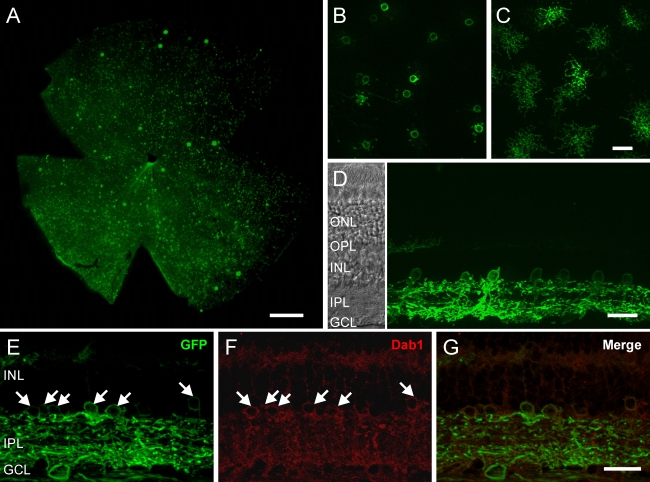
Predominant expression of Chop2-GFP in AII amacrine cells. **A**: In the retinal whole-mount, GFP-positive AII amacrine cells were found throughout the retina. **B**, **C**: At high magnification in the retinal whole-mount, GFP expression was detected in the somas of AII cells in the INL (**B**) and in the arboreal dendrites in the IPL (**C**). **D**: In the retinal vertical section, many AII amacrine cells were GFP-positive. **E-G**: In the same retina shown in **D**, GFP-positive cells (**E**) were immunolabeled for Disabled-1 (**F**). The merge image is shown in **G**. Double-labeled AII amacrine cells are marked by arrows. For **A**-**C**, GFP fluorescence was enhanced with antibodies against GFP. Abbreviations: outer nuclear layer (ONL); outer plexiform layer (OPL); inner nuclear layer (INL); inner plexiform layer (IPL); ganglion cell layer (GCL). Scale bars equal 500 μm in **A**, and 25 μm in **B**-**D**.

Interestingly, when the eyes were injected with a low concentration of virus, the GFP immunofluorescence was predominantly observed in AII amacrine cells. Figure 4 shows the Chop2-GFP expression pattern at the virus concentration of 1×10^10^ GC/ml. The bright puncta are the individual dendritic trees of AII amacrine cells (Figure 4A). Few ganglion cell axons can be seen around the blind spot. At higher magnification, numerous somas of presumably AII amacrine cells were visualized in the inner part of the INL (Figure 4B), and their dendrites were stratified in the ON-sublamina of the IPL (Figure 4C). In the vertical retinal section (Figure 4D), many GFP-immunofluorescent amacrine cells were encountered. We immunostained the same retina for Dab1 (Figure 4E), a known marker of AII amacrine cells [17]. All GFP-expressing amacrine cells were labeled and identified as AII amacrine cells. The density of GFP-expressing AII amacrine cells was 225±25 cells/mm^2^ (n=3). It is noteworthy that with a high virus concentration (6×10^12^ GC/ml), the majority of the GFP-expressing AII amacrine in the INL were also found to be AII amacrine cells based on immunostaining for Dab1 (data not shown). The density of the GFP-expressing AII amacrine for the latter was estimated to be 1,133±336 cells/mm^2^ (n=1).

**Figure 5:**
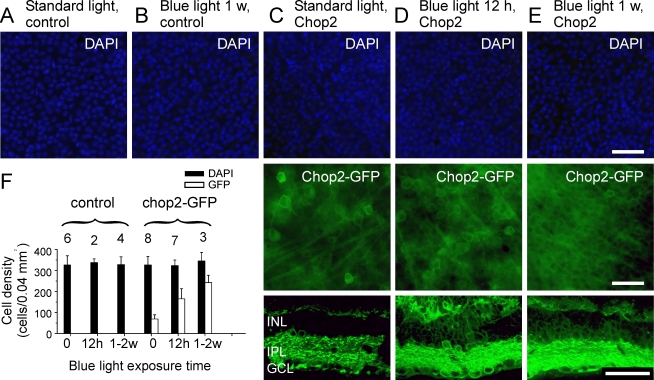
Comparison of cell densities under different light conditions. **A**-**B**: Representative images in whole-mount show the DAPI staining of the cells located in the GCL of the retinas for the un-injected animals that were kept under standard light conditions (**A**) or exposed for 1 week to the continuous strong blue light (**B**). **C-E**: Representative images show the virus-infected retinas form the animals that were exposed to standard light (**C**), or to a strong blue light for 12 h (**D**) and 1 week (**E**). Upper panels show the DAPI staining of the cells located in GCL. The middle panels show the GFP expression of the cells located in the GCL viewed in whole-mount. The low panels show the GFP expression viewed in vertical sections. **F**: Cell densities (black) and the number of GFP-positive cells (white) in the GCL from the control and virus-infected retinas were compared. The digits above the error bars indicate the number of studied retinas. Scale bars 50 μm.

The light illumination of the room that housed the animals measured at a wavelength of 500 nm was 6×10^14^ photons/cm^−2^s^−1^, and thus it might have been too low to significantly activate ChR2 channels [1]. Therefore, we asked whether neurotoxicity could be raised under light conditions in which the ChR2 channels are constantly activated. To address this question, in a group of mice as depicted in Figure 5, one eye was injected with the virus at the concentration of 6×10^12^ GC/ml and the second eye was left un-injected to serve as a control. After one month, the animals were exposed to a blue light for 12 h, 1 week, or 2 weeks (see Methods). The retinas of these mice were examined two weeks after the last day of the blue light exposure. The densities of the DAPI-labeled cells in the GCL were compared between the control retinas (Figure 5A,B) and the virus-infected retinas (Figure 5C-E; upper panels). The cell densities were similar (Figure 5F; *t*-test, p=0.8) in the retinas of the control animals kept under the standard light conditions (Figure 5A) to those exposed to the blue light (Figure 5B). Thus, the blue light itself did not cause any detectable damage to the cells in the GCL. Furthermore, no significant difference was found in the densities of the cells in the virus-injected retinas of mice between those under normal light conditions and those that were exposed to the blue light for 12 h, 1 week, or 2 weeks (Figure 5C-E; *t*-test, p=0.5–0.6). Interestingly however, the percentage of GFP-positive cells was significantly higher (*t*-test, p<0.01, Figure 5F) in the retinas exposed to blue light (Figure 5D,E, middle panels) than the control (Figure 5C, middle panel). Remarkably, an average of 51%±14% of the cells in the GCL expressed GFP after only a 12 h exposure to the blue light. After one or two weeks exposure to the blue light, the number of GFP-positive cells increased to 70%±10%, although the data between 12 h, 1 week, and 2 weeks were not statistically different (*t*-test, p=0.08). In the vertical sections taken with the same exposure time, the GFP fluorescence was much stronger in the retinas exposed to the blue light (Figure 5D,E, low panels) than in the control (Figure 5C, low panel). The dramatic increase of the GFP-positive cells and expression level is likely caused by the constant activation of ChR2 channels (see Discussion). Our results suggest that Chop2-GFP expressing retinal neurons are able to tolerate the prolonged activation of ChR2 in vivo in the eye.

### Physiological properties of ChR2 channels during the long-term expression

**Figure 6:**
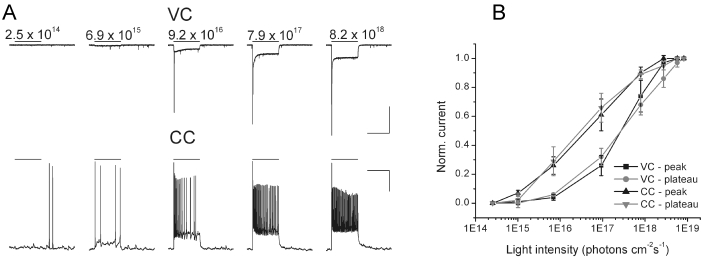
Physiological properties of ChR2 channels during long-term expression in mouse ganglion cells. **A**: The cell was held at V_hold_=−60 mV in voltage-clamp mode (VC, upper panel). ChR2-currents were elicited by light stimuli (Nicon B-3A fluorescence filter) with light intensities ranging from 2.5×10^14^ to 8.2×10^18^ photons cm^−2^s^−1^. During the same experiment in the current-clamp mode (CC, lower panel), the cell was held at −60 mV by I_hold_=−10 pA current injection. **B**: Normalized currents were plotted against light intensity. In VC, the half-maximal peak current was reached at 2.1×10^17^ photons cm^−2^s^−1^ and in CC the half-maximal peak potential at 3.2×10^16^ photons cm^−2^s^−1^. Scale bars 1 s and 300 pA for VC and 1 s and 20 mV for CC.

In this set of experiments, the functional properties of the ChR2 channels were examined in the retinas of two 20-month-old mice that were infected at the age of 2 months. Whole-cell patch-clamp recordings were performed on ganglion cells in vertical retinal slices. The precursor for the Chop2 chromophore group, all-trans retinal, was not added [1]. The light-evoked responses were observed in all recorded GFP-positive cells (n=10) but not in GFP-negative cells (n=3). The kinetics of the light-elicited responses was similar to that of ChR2 channels described previously [1]. The magnitude of the light-evoked responses was dependent on the light intensity (Figure 6A). It should note that, the initial transient current revealed in the voltage-clamp contains a mixture of two components. One is mediated by ChR2 channels and the other by voltage-gated sodium channels that have escaped from the voltage-clamp. The maximal amplitude of ChR2-mediated currents was −218±198 pA (measured at V_m_=-60 mV; n=7). This current was sufficient to drive the membrane depolarization and spike firing (Figure 6A, lower panel). The membrane potentials and the frequencies of spikes increased with an increase in the light intensity. Light-intensity response curves for the peak and plateau amplitudes are shown in Figure 6B.

**Figure 7:**
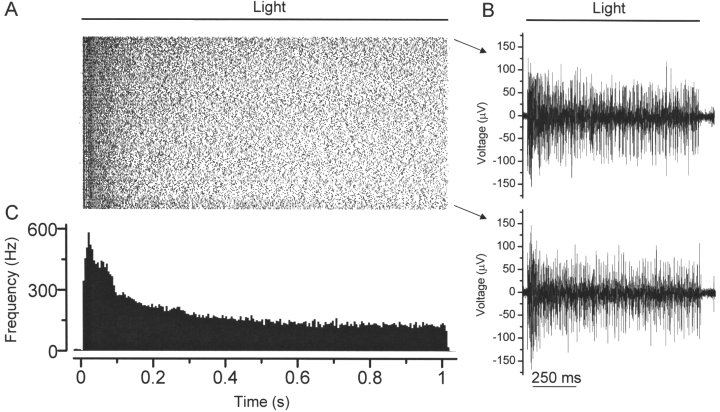
Multi-electrode array recordings from the retina 18 months after the viral injection. **A**: The raster plots consist of 180 consecutive light-elicited spike activities from a retinal ganglion cell during the course of a 3 h recording session. **B**: Sample light-evoked spikes were recorded at the beginning (top) and the end (bottom) of the recordings. **C**: The averaged spike rate was plotted versus the time. The duration of the light stimulation was 1 s and the interval between successive light stimuli was 60 s. The light intensity was 6.6×10^16^ photons cm^−2^s^−1^.

Finally, the ChR2-mediated responses were assessed by using multi-electrode array recording in whole-mount retinas. The recordings were performed in the presence of AMPA/kinate receptor antagonist CNQX (25 μM), NMDA receptor antagonist D-APV (25 μM), and mGluR6 receptor agonist L-APB (5 μM) to block potential photoreceptor cell-mediated light responses. Figure 7 shows the recordings of the ChR2-mediated spike firing from a retinal ganglion cell that was picked up by a single recording electrode. The spiking frequency was remarkably stable as demonstrated by the spike raster plot during a 3 h recording session (Figure 7A). The raw traces of the spike activity at the beginning and the end of the recording are shown in Figure 7B. The averaged spike rate histogram is shown in Figure 7C. Taken together, these results demonstrate the long-term stability of functional ChR2 channels in retinal neurons.

## Discussion

Virus-based gene delivery offers a promising therapeutic approach to restore the function of the retina in retinal degenerative diseases [18]. We used rAAV to deliver a fusion construct of Chop2 and GFP because of its high efficiency of transfecting retinal neurons and its ability to achieve long-term expression of transgenes [19-21]. The safety of rAAV vectors for retinal gene transfer has been successfully determined in animals [22]. Furthermore, clinical trials using rAAV vectors to treat retinal degenerative diseases, such as Leber congenital amaurosis, are currently under way [23]. GFP was used as a marker in our virus construct. The potential neurotoxicity of GFP in the retina has been assessed in a GFP-transgenic mouse line by Nour and colleagues [24]. Neither retinal function nor histology was affected by continuous bright light exposure (3500 lux). Moreover, GFP did not accelerate the course of retinal degeneration in the rhodopsin mouse model in the same study.

In this study, we expanded upon previous results and quantitatively examined the stability and safety of long-term expression of ChR2 channels in the mouse retinal neurons under different experimental conditions. These include virus concentrations, light conditions, and the expression duration of up to 18 months. Our results demonstrate the long-term stability and safety of ChR2-expression in retinal neurons. First, under our normal experimental conditions, about 20% of the neurons in the GCL were transfected by the virus vectors with highest concentration tested in this study. The percentage of Chop2-GFP-positive cells did not change during the examined period, and ChR2-expressing cells maintained their normal morphology.

Furthermore, no neurotoxicity was observed in the GCL in the virus-injected retinas, as assessed by the cell density in the GCL under normal light conditions and after exposure to a strong blue light for up to two weeks. However, the latter experiment may raise the question as to whether the blue light used in our experiments was strong enough to activate ChR2 in the retina of living animals. Two pieces of evidence suggest this is the case. First, the light intensity measured at the eye level of the animals in this study was 1.2×10^16^ photons cm^−2^s^−1^. This light intensity is about four times higher than what Lagali and colleagues used in their animal behavioral study [2]. In that experiment, rd1 mice expressing Chop2-GFP in retinal bipolar cells had a restored optomotor response when assessed by an array of blue LEDs at a light intensity of roughly 3×10^15^ photons cm^–2^s^–1^. Second, we found that the percentage of the ChR2-GFP positive cells as well as the GFP fluorescence in the light-exposed eyes was dramatically increased. The underlying mechanisms that cause this increase of ChR2-GFP expression remain to be studied. Activation of ChR2 can cause calcium influx through its channel or indirectly by activating voltage-gated calcium channels [6]. Elevated intracellular calcium can lead to the activation of transcriptional factors [25-27]. Therefore, it is possible that ChR2 expression was elevated after bright light exposure. The cells with fluorescence too low to be detected under the microscope expressed more ChR2-GFP and became visible. Hence there was an increase in the number of the counted GFP-positive cells. Whatever the mechanism, this observed phenomenon provided strong evidence that the bright blue light was sufficient to activate the ChR2 channels under our experimental conditions.

Our physiological recordings further demonstrate that a sufficient number of functional ChR2 channels are maintained in ganglion cells up to 18 months after a single viral vector administration. The channels were capable of driving robust and reliable spike firing in response to repeated light for several hours. Our study thus demonstrates the long-term stability of functional ChR2 in mammalian retinal neurons in vivo.

Finally, we show in this study that AAV vectors at a low virus concentration (1×10^10^ GC/ml) can predominantly infect AII amacrine cells. A significant number of AII amacrine cells were found to be infected. Rice and Curran estimated a density of 2,583 cells/mm^2^ of AII amacrine cells that were labeled with the Disabled-1 antibody [17]. In our study, 225±25 cells/mm^2^ (n=3) were labeled with GFP, which corresponds to roughly 9% of the entire AII cell population. In addition, at a high virus concentration (6×10^12^ GC/ml), the majority of the Chop2-GFP-expressing amacrine cells in the INL were found to be AII amacrine cells. The density of infected AII amacrine cells under the latter condition was estimated to be 1,133±366 cells/mm^2^, which is roughly 40% of all AII amacrine cells. Our results, therefore, indicated that rAAV2 vectors with CAG promoter are able to preferably target to AII amacrine cells in the mouse retina. The underlying mechanism of targeting to AII amacrine cells is also interesting. Since the bodies of AII amacrine cells are located in the INL, the virus must be able to diffuse deep inside the tissue, thereby surpassing the GCL. Because we used a ubiquitous promoter in this study, therefore it is likely that the AII amacrine cells have high affinity receptors for this virus serotype [28]. Also, these receptors could be located on the arboreal dendrites that are close to the intravitreal space where the virus vectors were injected. The retrograde transport of rAAV from the distant processes to the nucleus of the infected cells has been previously reported [29-31].

In mammals, including humans, the rod signal is relayed through rod bipolar cells to AII amacrine cells and then piggybacked onto ON and OFF cone pathways by gap junctions and glycinergic synapses, respectively [32]. Thus, the ability to target ChR2 to AII amacrine cells provides a potential approach to restore both ON and OFF light responses in the degenerated retina. Further studies will need to determine whether the virus concentration dependent selectivity would hold in other species, including humans. In addition, the ability to target transgenes to AII amacrine cells offers a useful tool for studying the physiologic functions as well as manipulating the molecular properties of this particular cell type.
